# Supplementary material for: Proliferation associated 2G4 is required for the ciliation of vertebrate motile cilia
Source: Commun Biol. 2024 Nov 4;7:1430. doi: 10.1038/s42003-024-07150-0 (PMC11535434; doi:10.1038/s42003-024-07150-0)

Supplementary information

Proliferation associated 2G4 is required for the ciliation of vertebrate motile cilia

Moonsup Lee[1], Christina Carpenter[2], Yoo-Seok Hwang[1], Jaeho Yoon[1], Quanlong Lu[3], Christopher J. Westlake[3], Sally A. Moody[4], Terry P. Yamaguchi[1,*], and Ira O. Daar[1,*]

[1] Cancer & Developmental Biology Laboratory, Center for Cancer Research, National Cancer Institute, National Institutes of Health, Frederick, MD 21702, USA

[2] Electron Microscopy Laboratory, Frederick National Laboratory for Cancer Research, Frederick, MD, USA

[3] Laboratory of Cell and Developmental Signaling, Center for Cancer Research, National Cancer Institute, National Institutes of Health, Frederick, MD 21702, USA

[4] Department of Anatomy and Cell Biology, George Washington University, School of Medicine and Health Sciences, Washington, DC, USA

Supplementary Fig. 1

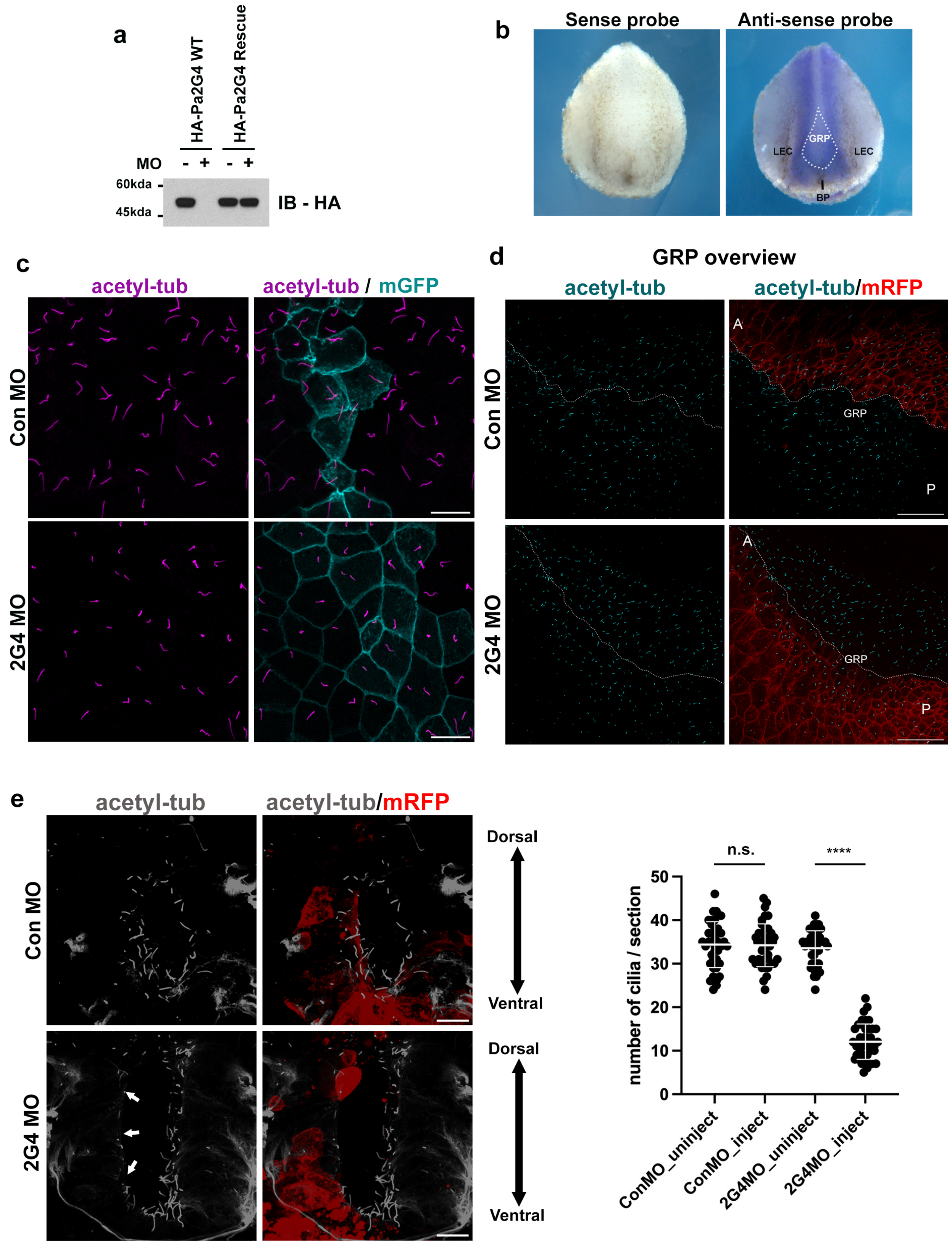

Supplementary Figure 1. Pa2G4 is required for ciliogenesis in GRP cells.

(a) the inhibitory effect of 2G4MO using exogenous HA-tagged Pa2G4. WB analysis showed the 2G4 MO specifically decreased wild-type HA-Pa2G4 expression, but HA-Pa2G4 rescue (MO-resistant Pa2G4 mRNA with 6 nucleotide mutations in the MO binding region) remained unaffected by 2G4 MO. The embryos were microinjected with the indicated mRNAs and MOs and harvested at stage 12 for immuno-blot. (b) Pa2G4 is widely expressed in the GRP region. Whole-mount in situ hybridization was conducted using a neurula stage embryo (stage 18). LEC, lateral endodermal cells; bp, blastopore. (c and d) Pa2G4 knockdown shortened GRP cilia. The indicated mRNA and MOs were injected into two dorsal marginal zones at eight-cell stage embryos. mem-GFP (cyan; panel (c)) and mem-RFP (red; panel (d)) were used as a tracer. anti-acetylated tubulin antibody stains mono-motile cilia (magenta; panel (c), cyan; panel (d)) in the GRP. Scale bars, 20µm (panel c), 100µm (panel d). (e) Pa2G4 is required for primary cilia formation in the neural tube. Pa2G4 MOs were injected into one dorsal blastomere of 8-cell stage embryos to target the neural tube, and the embryos at stage 25 were transversely sectioned, followed by staining with acetylated tubulin (gray). mem-RFP was used as a tracer. White arrows indicate reduced acetylated tubulin signals. Scale bars, 20µm. Quantification of cilia number per section, $n = 40$; embryos per group $n = 10$; unpaired two-tailed Student's t-test, ****, $p < 0.0001$; error bars represent SD.

Supplementary Fig. 2

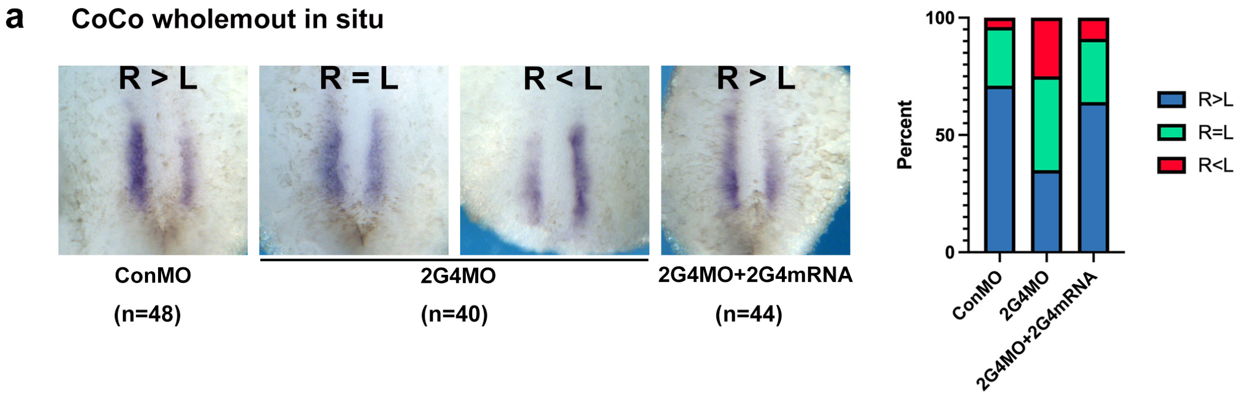

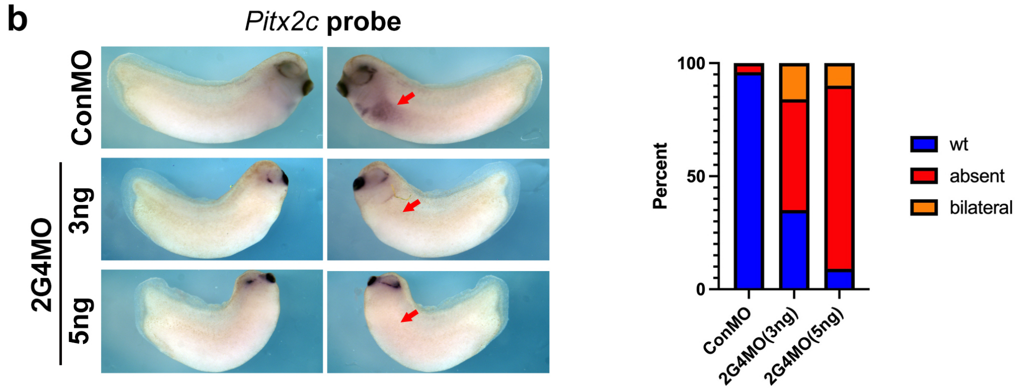

Supplementary Figure 2. Pa2G4 knockdown disturbs a leftward flow in the GRP

(a) The representative Coco mRNA expression pattern at stages 20-21 was disturbed by Pa2G4 knockdown. Wholemount in situ hybridization was conducted with a Coco probe. R > L image from control, R = L, and R < L images from Pa2G4 morphants. The number of embryos for quantification: control, n = 24; Pa2G4 MO, n = 20; rescue, n = 22. (b) whole-mount in situ hybridization using the *pitx2c* probe. Control morphants (50/52) showed wild-type expression in the left-lateral plate mesoderm. However, as the red arrows point out, depending on MO concentration, most 2G4 morphants lacked pitx2c expression (2G4MO(3ng); 30/61, 2G4MO(5ng); 50/58) and only a small portion of 2G4 morphants showed bilateral expression (2G4MO(3ng); 5/61, 2G4MO(5ng); 2/58).

Supplementary Fig. 3

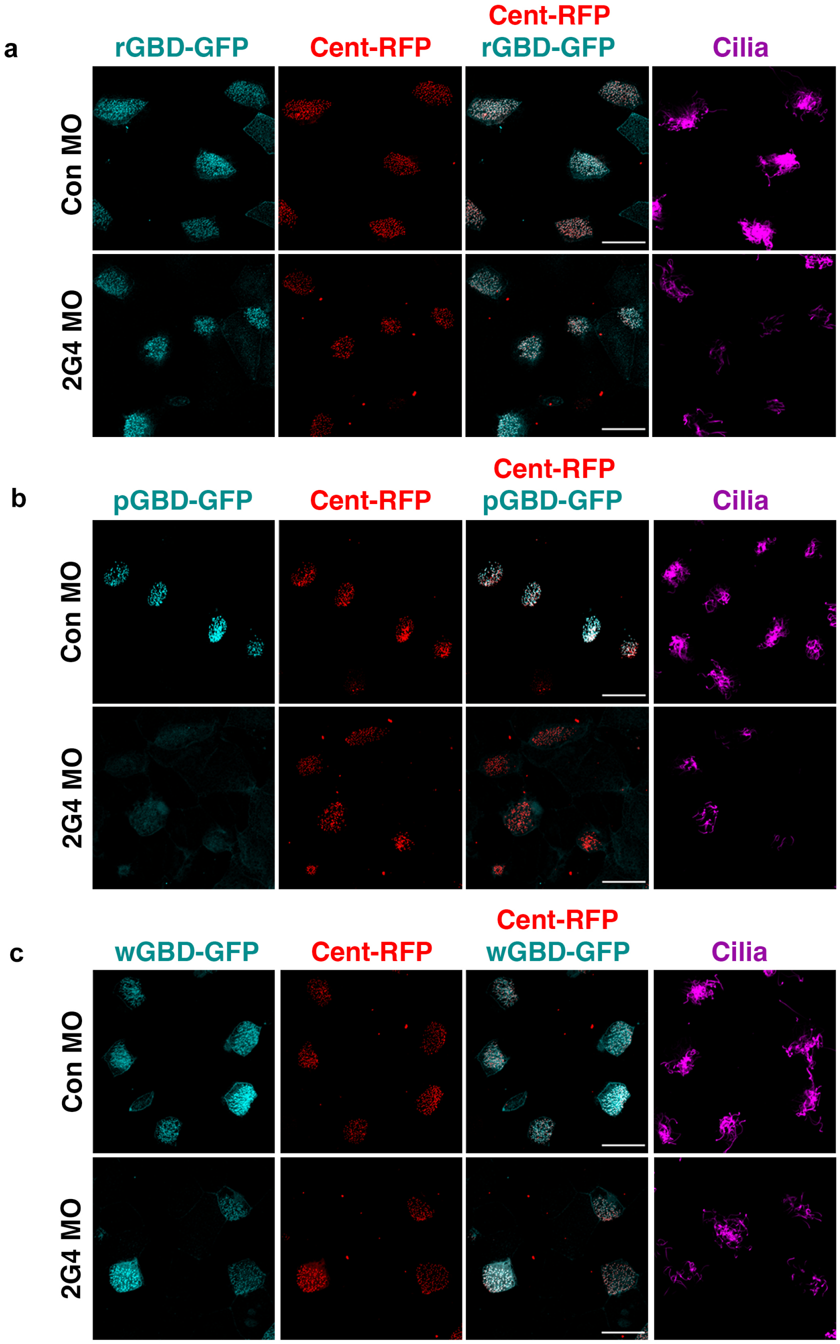

Supplementary Figure 3. Pa2G4 knockdown reduces Rac1 activity but not RhoA and Cdc42 in MCCs. (a-c) the indicated mRNAs of rGBD-GFP (a), pGBD-GFP (b), and wGBD-GFP (c) were co-injected with centrin-RFP mRNA and MOs to one ventral blastomere at eight-cell stage embryos. Anti-acetylated tubulin staining represents multi-cilia (magenta). Scale bars, 20µm.

Supplementary Fig. 4

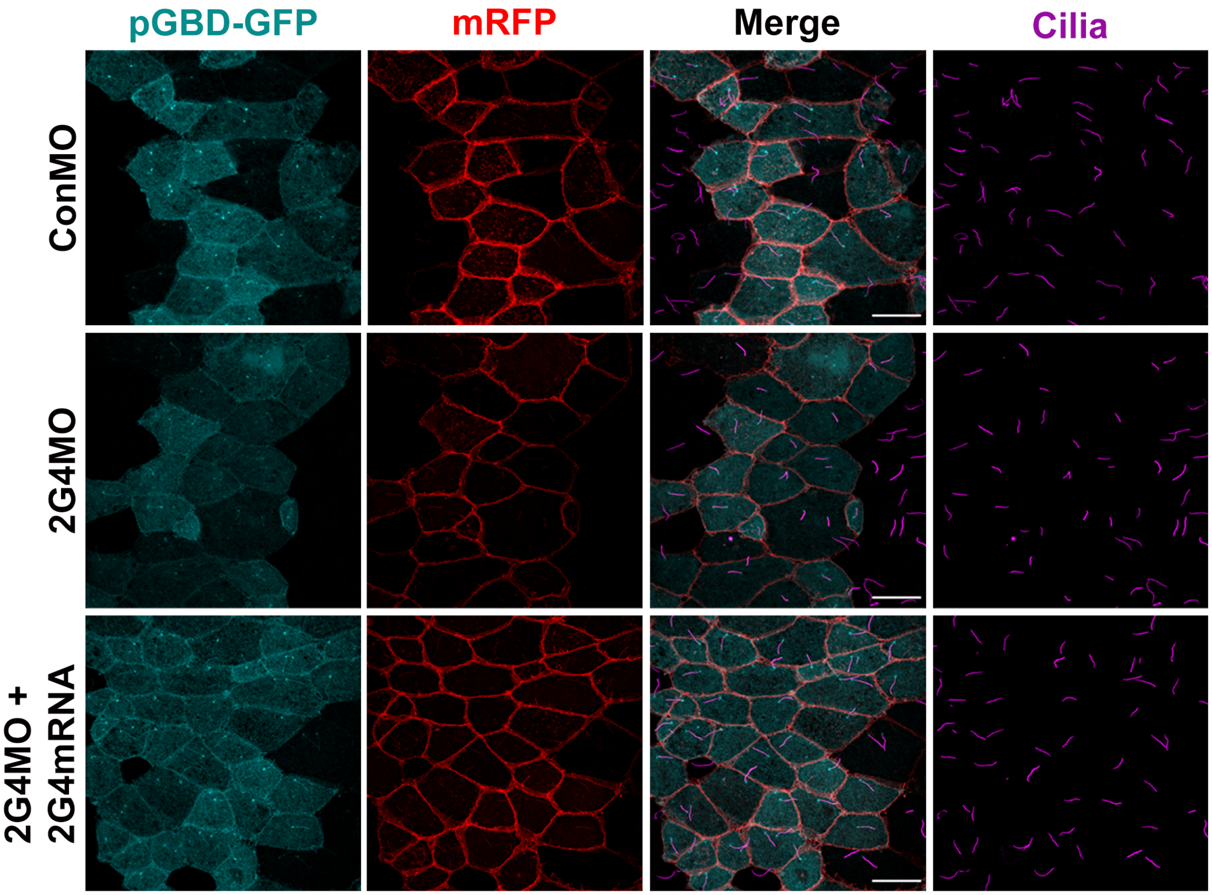

Supplementary Figure 4. Rac1 activity decreases in Pa2G4 morphant GRP cells.

The indicated mRNAs and MOs were injected into two dorsal blastomeres at eight-cell stage embryos, and embryos were dissected at stage 17 for immunostaining. pGBD-GFP (cyan), mRFP (red, tracer), Cilia (magenta, marked by anti-acetylated tubulin antibody). Scale bars, 20μm.

Supplementary Fig. 5

a

```
H. Sapiens NP_006182.2         MSGEDEQQEQTIAEDLVVTKYKMGGDIANRVLRSLVEASSSGVSVLSLCEKGDAMIMEET
X. laevis  NP_001085830.1      MSGDEEQQEQTIAEDLVVTKYKMGGDIANRVLRTLLDTATAGASLLNLCEKGDAMIMEET
                               ***::****************************:*::::::*.*:*.*************

           NP_006182.2         GKIFKKEKEMKKGIAFPTSISVNNCVCHFSPLKSDQDYILKEGDLVKIDLGVHVDGFIAN
           NP_001085830.1      GKIFKKEKEMKKGIAFPTSISVNNCVCHFSPLKSDQDYLLKDGDLVKIDLGVHVDGFIAN
                               **************************************:**:******************

           NP_006182.2         VAHTFVVDVAQGTQVTGRKADVIKAAHLCAEAALRLVKPGNQNTQVTEAWNKVAHSFNCT
           NP_001085830.1      VAHSFVVGASKECPVTGRKADVIKAAHLCGEAALRLVKPGNQNTQVTEAWNKISPEFKCT
                               ***:***..::   ***************.**********************:: .*:**

           NP_006182.2         PIEGMLSHQLKQHVIDGEKTIIQNPTDQQKKDHEKAEFEVHEVYAVDVLVSSGEGKAKDA
           NP_001085830.1      PIEGMLSHQLKQHVIDGEKTIIQNPTDQQKKDHEKAEFEVHEVYAVDVLISTGEGKAKDA
                               *************************************************:*:********

           NP_006182.2         GQRTTIYKRDPSKQYGLKMKTSRAFFSEVERRFDAMPFTLRAFEDEKKARMGVVECAKHE
           NP_001085830.1      GQRTTIYKRDPTKQYGLKMKTSRAFFSEVERRFDAMPFTLRAFEDEKKARMGVVECAKHE
                               ***********:************************************************

           NP_006182.2         LLQPFNVLYEKEGEFVAQFKFTVLLMPNGPMRITSGPFEPDLYKSEMEVQDAELKALLQS
           NP_001085830.1      LLQPFNVLYEKEGEFVAQFKFTVLLMPNGPMRITSGPFEPDLYKSELEVQDSELKALLQS
                               **********************************************:****:********

           NP_006182.2         SASRKTQKKKKKASKTAENATSGETLEENEAGD
           NP_001085830.1      SASRKTQKKKKKASKTAENATAEENEGTE----
                               *********************: *.   :
```

b

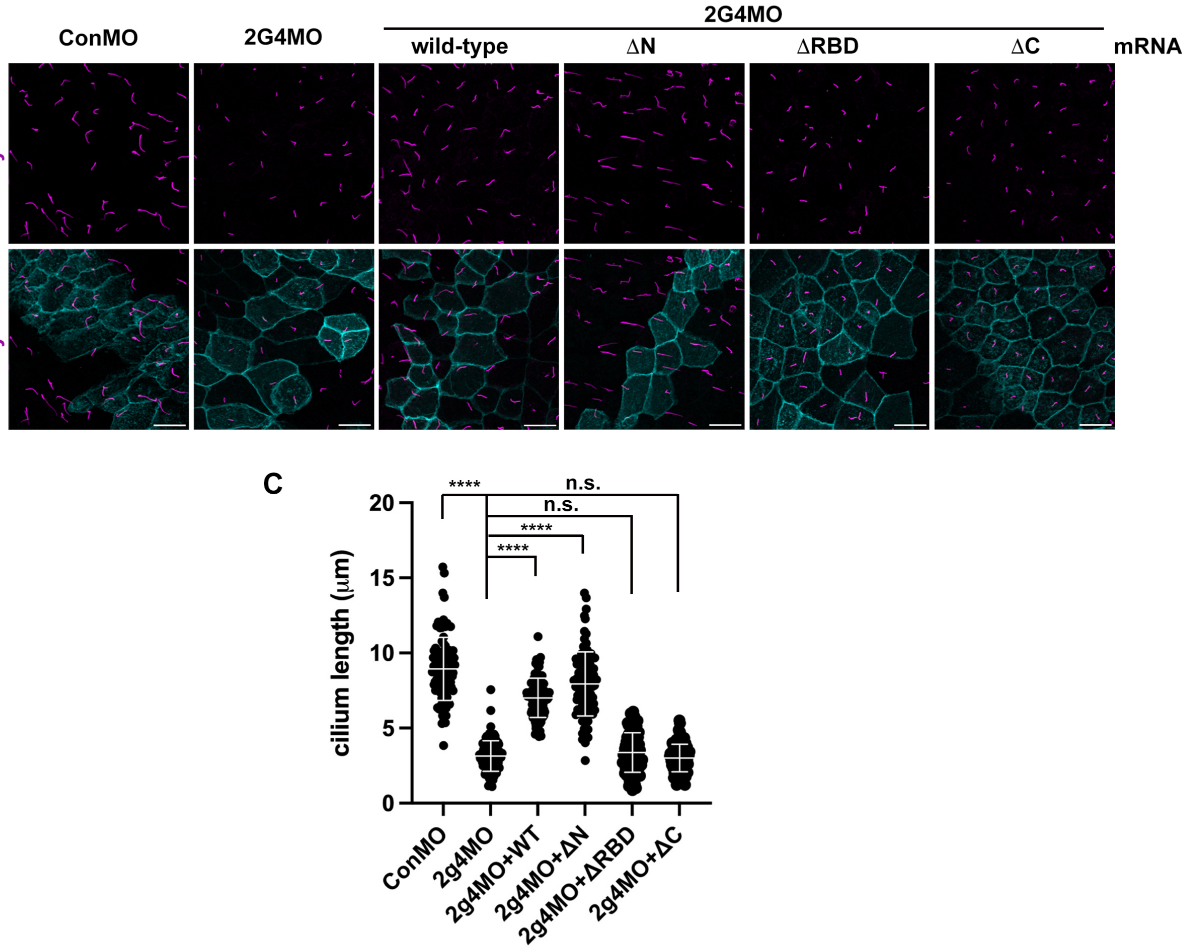

Supplementary Figure 5. The RBD and C-terminal regions of Pa2G4 are required for ciliogenesis in GRP cells.

(a) amino acid sequence comparison between human Pa2G4 and *Xenopus laevis* Pa2G4. (b) ΔRBD and ΔC did not rescue shortened GRP cilia upon Pa2G4 knockdown. acetylated tubulin staining marks cilia. mem-GFP is used as a tracer. (c) Quantification of GRP cilia length in (b). cilia number per group, $n > 100$; dissected GRPs per group from three independent experiments, $n = 10$. ****, $P < 0.0001$; one-way ANOVA. Error bars mean ±SD.

Supplementary Fig. 6

original data for Figure 5d

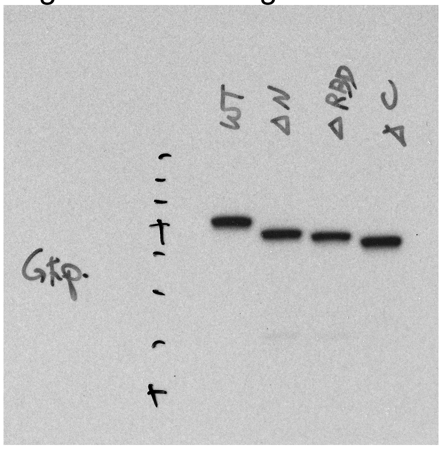

original data for Supplementary Figure 1a

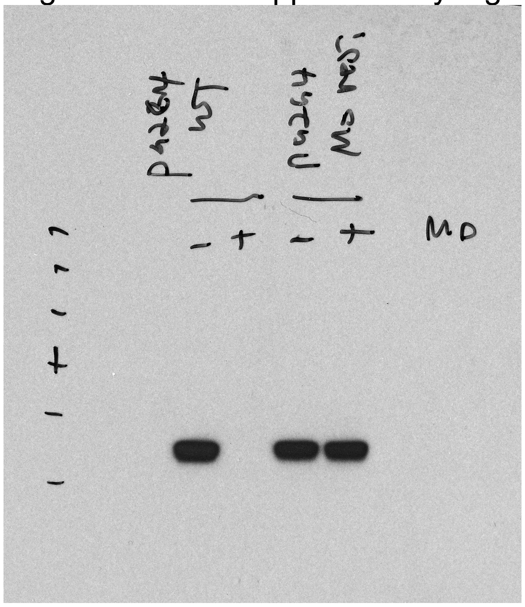

Supplement: Supplementary file 2 — supplementary information [file 42003_2024_7150_MOESM2_ESM.pdf]
