# Supplementary material for: Proliferation associated 2G4 is required for the ciliation of vertebrate motile cilia
Source: Commun Biol. 2024 Nov 4;7:1430. doi: 10.1038/s42003-024-07150-0 (PMC11535434; doi:10.1038/s42003-024-07150-0)
Supplement: Supplementary file 3 — Description of Additional Supplementary Files [file 42003_2024_7150_MOESM3_ESM.pdf]

Description of Additional Supplementary Files

File name: Supplementary Data

Description: The source data behind the graphs in the paper.

File name: Supplementary Movie 1

Description: Multi-cilia beating in representative control morphant. Frame rate 300 frames/second.

File name: Supplementary Movie 2

Description: Multi-cilia beating in representative Pa2G4 morphant. Frame rate 300 frames/second.

File name: Supplementary Movie 3

Description: Multi-cilia beating in representative Rescue embryo. Frame rate 300 frames/second.
