# Supplementary material for: Proliferation associated 2G4 is required for the ciliation of vertebrate motile cilia
Source: Commun Biol. 2024 Nov 4;7:1430. doi: 10.1038/s42003-024-07150-0 (PMC11535434; doi:10.1038/s42003-024-07150-0)
Supplement: Supplementary file 8 — reporting summary [file 42003_2024_7150_MOESM8_ESM.pdf]

nature portfolio

Corresponding author(s): Ira O. Daar and Terry P. Yamaguchi

Last updated by author(s): Oct 22, 2024

Reporting Summary

Nature Portfolio wishes to improve the reproducibility of the work that we publish. This form provides structure for consistency and transparency in reporting. For further information on Nature Portfolio policies, see our Editorial Policies and the Editorial Policy Checklist.

Statistics

For all statistical analyses, confirm that the following items are present in the figure legend, table legend, main text, or Methods section.

n/a	Confirmed	
☐	☒	The exact sample size (*n*) for each experimental group/condition, given as a discrete number and unit of measurement
☐	☒	A statement on whether measurements were taken from distinct samples or whether the same sample was measured repeatedly
☐	☒	The statistical test(s) used AND whether they are one- or two-sided<br>*Only common tests should be described solely by name; describe more complex techniques in the Methods section.*
☐	☒	A description of all covariates tested
☐	☒	A description of any assumptions or corrections, such as tests of normality and adjustment for multiple comparisons
☐	☒	A full description of the statistical parameters including central tendency (e.g. means) or other basic estimates (e.g. regression coefficient) AND variation (e.g. standard deviation) or associated estimates of uncertainty (e.g. confidence intervals)
☒	☐	For null hypothesis testing, the test statistic (e.g. *F*, *t*, *r*) with confidence intervals, effect sizes, degrees of freedom and *P* value noted<br>*Give P values as exact values whenever suitable.*
☒	☐	For Bayesian analysis, information on the choice of priors and Markov chain Monte Carlo settings
☒	☐	For hierarchical and complex designs, identification of the appropriate level for tests and full reporting of outcomes
☒	☐	Estimates of effect sizes (e.g. Cohen's *d*, Pearson's *r*), indicating how they were calculated

Our web collection on statistics for biologists contains articles on many of the points above.

Software and code

Policy information about availability of computer code

Data collection	ImageJ program was used for all Data collection.
Data analysis	Prism10 program was used for all statistical analysis.

For manuscripts utilizing custom algorithms or software that are central to the research but not yet described in published literature, software must be made available to editors and reviewers. We strongly encourage code deposition in a community repository (e.g. GitHub). See the Nature Portfolio guidelines for submitting code & software for further information.

Data

Policy information about availability of data

All manuscripts must include a data availability statement. This statement should provide the following information, where applicable:

- Accession codes, unique identifiers, or web links for publicly available datasets
- A description of any restrictions on data availability
- For clinical datasets or third party data, please ensure that the statement adheres to our policy

Stated in the methods section, Imaging data and other data supporting the findings in this study are available from the corresponding author on reasonable request.

Research involving human participants, their data, or biological material

Policy information about studies with human participants or human data. See also policy information about sex, gender (identity/presentation), and sexual orientation and race, ethnicity and racism.

Reporting on sex and gender	No human research samples were used in this study.
Reporting on race, ethnicity, or other socially relevant groupings	No human research samples were used in this study.
Population characteristics	No human research samples were used in this study.
Recruitment	No human research samples were used in this study.
Ethics oversight	No human research samples were used in this study.

Note that full information on the approval of the study protocol must also be provided in the manuscript.

Field-specific reporting

Please select the one below that is the best fit for your research. If you are not sure, read the appropriate sections before making your selection.

☒ Life sciences ☐ Behavioural & social sciences ☐ Ecological, evolutionary & environmental sciences

For a reference copy of the document with all sections, see nature.com/documents/nr-reporting-summary-flat.pdf

Life sciences study design

All studies must disclose on these points even when the disclosure is negative.

Sample size	Stated in the Material and Methods, Statistical analysis. The sample size was determined as indicated in the figure, and specific statistical method was used.
Data exclusions	Embryos which have mis-targeted injection were excluded (the target injection was confirmed by flourorescent-tagged morpholino or coinjection with fluorescent dextrans.
Replication	Stated in the Figure legends in each figure. At least three independent experiments were performed.
Randomization	Stated in the Material and Methods, Statistical analysis.
Blinding	the analyses were not blinded.

Reporting for specific materials, systems and methods

We require information from authors about some types of materials, experimental systems and methods used in many studies. Here, indicate whether each material, system or method listed is relevant to your study. If you are not sure if a list item applies to your research, read the appropriate section before selecting a response.

Materials & experimental systems

n/a	Involved in the study	
☐	☒	Antibodies
☒	☐	Eukaryotic cell lines
☒	☐	Palaeontology and archaeology
☐	☒	Animals and other organisms
☒	☐	Clinical data
☒	☐	Dual use research of concern
☒	☐	Plants

Methods

n/a	Involved in the study	
☒	☐	ChIP-seq
☒	☐	Flow cytometry
☒	☐	MRI-based neuroimaging

Antibodies

Antibodies used	HRP-conjugated anti-GFP antibody (dilution 1:2000, MA5-15256-HRP, ThermoFisher) anti-Digoxigennin-AP antibody (11093274910, Roche)<br>anti-acetylated tubulin (T7451; Sigma-Aldrich) chicken anti-GFP (NB100-1614; Novus Biologicals) anti-Cep164 (22227-1-AP; Protein

	Tech)<br>anti-centrin (04-1624; EMD Millipore)<br>Goat anti-Mouse IgG (H+L) Cross-Adsorbed Secondary Antibody, Alexa Fluor™ 647 (A-21235; ThermoFisher)<br>Goat anti-Rabbit IgG (H+L) Cross-Adsorbed Secondary Antibody, Alexa Fluor™ 555 (A-21428; ThermoFisher)<br>Goat anti-Rabbit IgG (H+L) Highly Cross-Adsorbed Secondary Antibody, Alexa Fluor™ 647 (A-21245: ThermoFisher)<br>Goat anti-Chichen IgY (H+L) Secondary Antibody,Alexa Fluor 488 (A-11039; ThermoFIsher)
Validation	Antibodies were validated by Wholemount in situ hybridization, Western blot analysis and Immunofluorescence microscopy in the presence or absence of antisense morpholinos.

Animals and other research organisms

Policy information about studies involving animals; ARRIVE guidelines recommended for reporting animal research, and Sex and Gender in Research

Laboratory animals	Xenopus laevis
Wild animals	Adults female Xenopus laevies (from NASCO, 1.5 year old), Adults male Xenopus laevies (from NASCO, 2 year old)
Reporting on sex	Adults female Xenopus laevies were used to obtain oocytes. Adults male Xenopus laevies were used to obtain sperm.
Field-collected samples	No field-collected samples were used in this study.
Ethics oversight	All animal studies were approved by the NCI at Frederick Animal Care and Use Committee [ACUC] under ASP #23-433.

Note that full information on the approval of the study protocol must also be provided in the manuscript.

Plants

Seed stocks	N/A
Novel plant genotypes	N/A
Authentication	N/A
